# Author Correction: A benchmark of computational methods for correcting biases of established and unknown origin in CRISPR-Cas9 screening data

**DOI:** 10.1186/s13059-024-03387-4

**Published:** 2024-09-04

**Authors:** Alessandro Vinceti, Rafaele M. Iannuzzi, Isabella Boyle, Lucia Trastulla, Catarina D. Campbell, Francisca Vazquez, Joshua M. Dempster, Francesco Iorio

**Affiliations:** 1 Computational Biology Research Centre, Human Technopole, Milan, Italy; 2 Broad Institute of Harvard and MIT, Cambridge, MA USA

**Correction** **:** **Genome Biol 25, 192 (2024)**

**https://doi.org/10.1186/s13059-024-03336-1**

Following publication of the original article [1], the authors identified an omission in the completing interests section. The omitted text is given in bold below.

**Competing interests**

FI receives funding from Open Targets, a public-private initiative involving academia and industry and performs consultancy for the joint CRUK-AstraZeneca Functional Genomics Centre and for Mosaic TX. JD is a consultant for and holds equity in Jumble Therapeutics. CDC performs consultancy for Droplet Biosciences and is a shareholder of Novartis. **FV receives research support from the Dependency Map Consortium, Riva Therapeutics, Bristol Myers Squibb, Merck, Illumina, and Deerfield Management. FV is on the scientific advisory board of GSK, is a consultant and holds equity in Riva Therapeutics and is a co-founder and holds equity in Jumble Therapeutics**. All other authors declare that they have no competing interests.

The original article [1] is corrected.

## References

[1] Vinceti A, Iannuzzi RM, Boyle I, et al. A benchmark of computational methods for correcting biases of established and unknown origin in CRISPR-Cas9 screening data. Genome Biol. 2024;25:192. 10.1186/s13059-024-03336-1. 39030569 10.1186/s13059-024-03336-1 PMC11264729

