# Supplementary material for: A benchmark of computational methods for correcting biases of established and unknown origin in CRISPR-Cas9 screening data
Source: Genome Biol. 2024 Jul 19;25:192. doi: 10.1186/s13059-024-03336-1 (PMC11264729; doi:10.1186/s13059-024-03336-1)
Supplement: Supplementary file 1 — Additional file 1. Supplementary Figures and Tables. [file 13059_2024_3336_MOESM1_ESM.pdf]

Supplementary Material for

A benchmark of computational methods for correcting biases of established and unknown origin in CRISPR-Cas9 screening data

Alessandro Vinceti[1], Raffaele Iannuzzi[1], Isabella Boyle[2], Lucia Trastulla[1], Catarina D. Campbell[2], Francisca Vazquez[2], Joshua Dempster[2], Francesco Iorio[1,*]

[1]Computational Biology Research Centre, Human Technopole - Milan, Italy
[2]Broad Institute of Harvard and MIT, Cambridge, MA, USA

[*]Corresponding author: francesco.iorio@fht.org

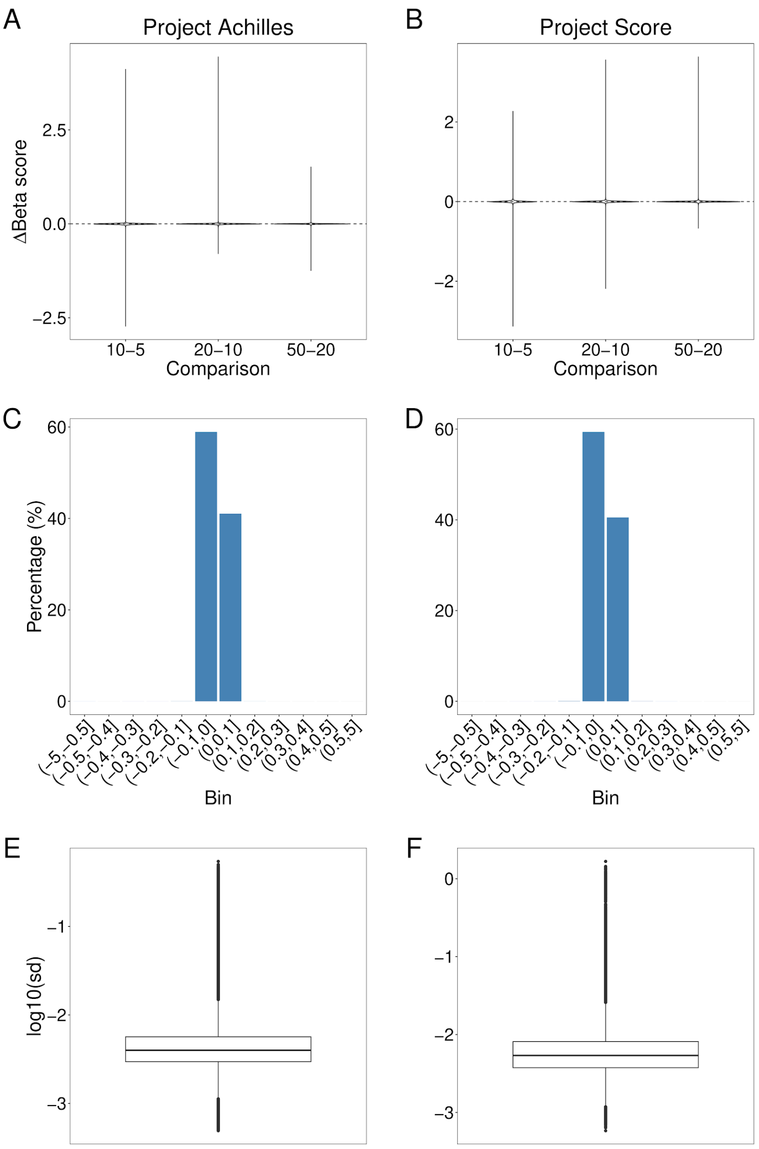

Fig. S1 - Impact of batching on MAGeCK's correction outcome. AB. Pointwise difference between MAGeCK-corrected Project Achilles (A) and Project Score (B) datasets processed in different batch sizes (i.e. 5, 10, 20 and 50 cell lines). CD. Binning of pointwise differences for Project Achilles (C) and Project Score (D) datasets, processed in batches of 20 and 50 cell lines. EF. Pointwise standard deviation across 100 MAGeCK-corrected Project Achilles (E) and Project Score (F) datasets, processed in batches of 50 cell lines, using different seed values.

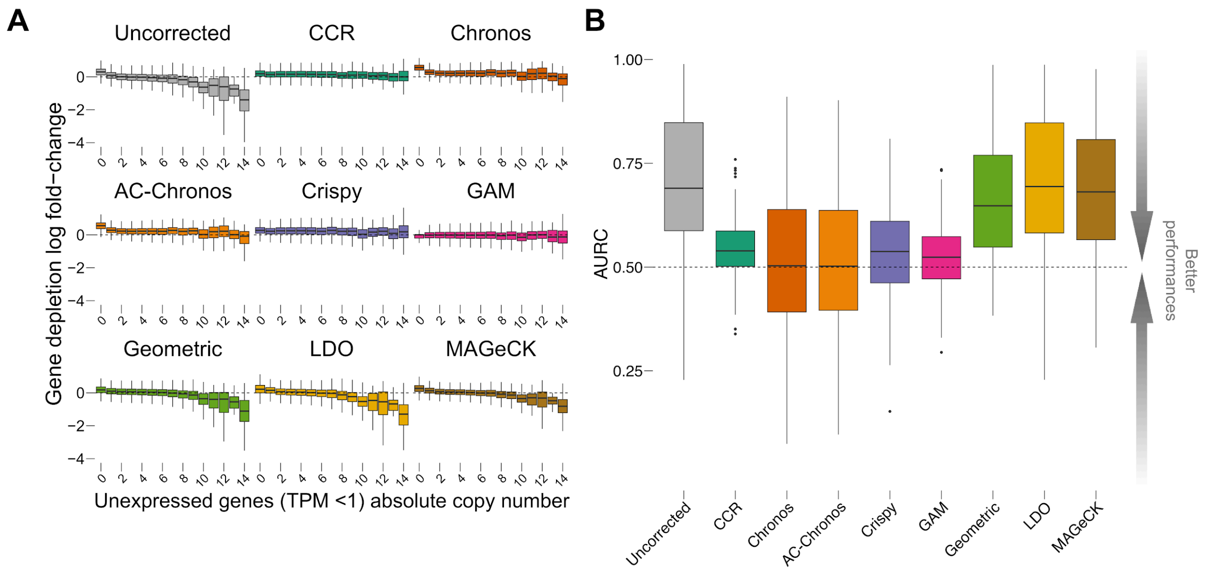

Fig. S2 - Correction of copy number bias for unexpressed genes in Project Score. A. Gene-level log fold-change (LFC) values of unexpressed genes (TPM < 1) on a per-cell line basis in the Project Score dataset grouped according to their absolute copy number value (using PICNIC data) before and after each method correction. B. For each method, we computed the cell-wise Area Under the Recall Curve (AURC) of the top 1% amplified unexpressed genes using the remaining unexpressed genes as outgroup.

Method	Project Achilles		Project Score	
	ARD	AURC (Top 1% amplified unexpressed genes)	ARD	AURC (Top 1% amplified unexpressed genes)
Uncorrected	0.66±0.828	0.701±0.13	0.508±0.454	0.703±0.16
CCR	0.305±0188	0.544±0.0711	0.341±0.19	0.543±0.0716
Chronos	0.829±0.498	0.487±0.137	0.698±0.473	**0.512±0.174**
AC-Chronos	0.794±0.455	**0.489±0.132**	0.667±0.451	0.516±0.167
Crispy	0.436±0.213	0.532±0.0915	0.552±0.237	0.531±0.108
GAM	**0.136±0.318**	0.515±0.07	**0.0908±0.107**	0.521±0.081
Geometric	0.507±0.666	0.645±0.126	0.381±0.333	0.669±0.14
LDO	0.628±0.794	0.695±0.128	0.462±0.401	0.698±0.159
MAGeCK	0.673±0.868	0.703±0.13	0.488±0.408	0.68±0.153

Table S1 - Summary of average residual divergence (ARD) and area under the recall curve (AURC) of copy number bias correction (mean ± standard deviation) for unexpressed genes across methods in Project Achilles and Project Score. Bold is the best outcome across methods.

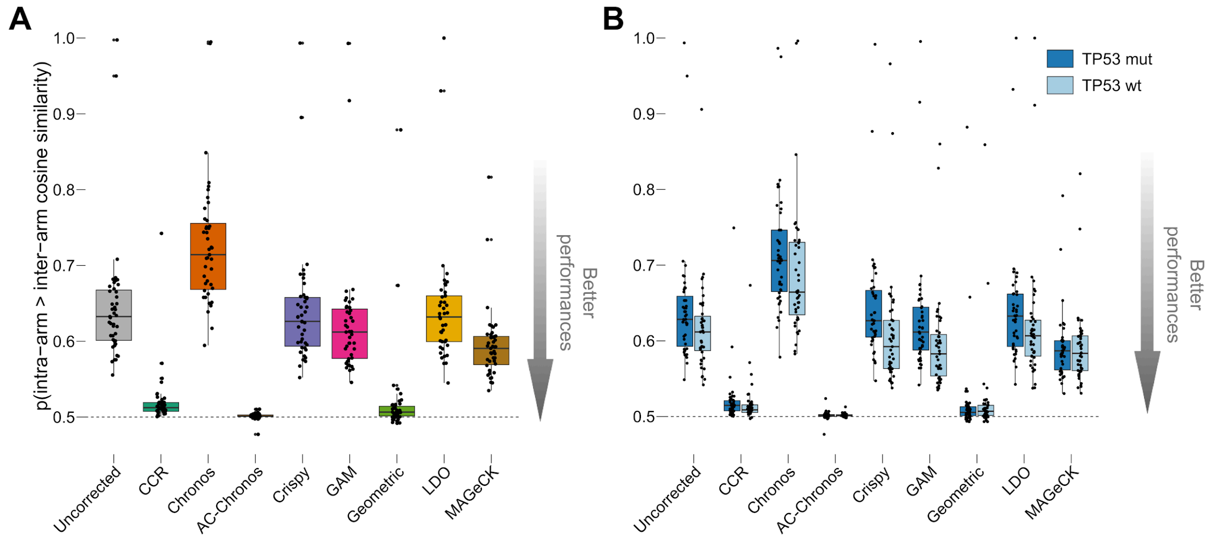

Fig. S3 - Correction of proximity bias in Project Score. A. Chromosome arm-level proximity bias quantified by the Brunner-Munzel test statistics for the Project Score dataset before (uncorrected) and after each method's correction. Each point is a chromosome arm. B. Chromosome arm-level proximity bias as in A. but from cell lines split based on the TP53 status. All boxplots represent distributions with central lines indicating median values and lower and upper whiskers encompassing scores within -1.5 and 1.5 times the interquartile range.

Method	GW	P-val	TP53 WT	P-val	TP53 LOF	P-val	AR
Uncorrected	0.628±0.0424	NA	0.627±0.0423	NA	0.618±0.0419	NA	1.015
CCR	0.52±0.0195	4.69e-21	0.523±0.0228	8.36e-20	0.516±0.0154	1.49e-20	1.014
Chronos	0.716±0.0599	5.59e-11	0.706±0.0587	2.35e-8	0.686±0.0566	1.09e-9	1.029
AC-Chronos	**0.501±0.00223**	8.56e-22	**0.501±0.00234**	1.29e-20	**0.501±0.00194**	1.2e-21	**0.9998**
Crispy	0.607±0.0402	0.025	0.619±0.0426	9.72e-4	0.588±0.036	4.34e-1	1.053
GAM	0.596±0.0389	6.85e-4	0.608±0.0412	1.48e-5	0.578±0.0349	4.61e-2	1.051
Geometric	0.508±0.0154	**1.52e-22**	0.508±0.0177	**6.54e-21**	0.507±0.0126	**9.98e-23**	**1.0002**
LDO	0.621±0.043	0.487	0.621±0.0436	4.65e-1	0.611±0.0421	5.52e-1	1.017
MAGeCK	0.635±0.0429	0.476	0.633±0.0419	4.62e-1	0.625±0.0427	5.13e-1	1.014

Table S2 - Summary of proximity number bias (mean ± standard deviation) correction across methods in Project Achilles. Results are shown both for genome-wide (GW) and TP53-based proximity bias (i.e. wild-type (WT) and loss-of-function (LOF)). For genome-wide and TP53-based proximity bias, we also performed a *t*-test to assess whether each method's distribution is significantly different from the uncorrected one. For the TP53-based proximity bias, we computed the average ratio (AR) between the WT and LOF proximity bias scores for each method. Bold is the best outcome across methods.

Method	GW	P-val	TP53 WT	P-val	TP53 LOF	P-val	AR
Uncorrected	0.646±0.0821	NA	0.643±0.0825	NA	0.624±0.0824	NA	1.032
CCR	0.521±0.0371	7.89e-13	0.522±0.0387	8.36e-20	0.517±0.0282	1.49e-20	1.009
Chronos	0.726±0.0822	2.1e-5	0.717±0.0809	2.35e-8	0.69±0.0882	1.09e-9	1.043
AC-Chronos	**0.501±0.0046**	**1.11e-14**	0.502±0.0023	2.38e-12	**0.502±0.0056**	2.66e-14	0.9992
Crispy	0.64±0.0787	0.721	0.646±0.0768	9.72e-4	0.609±0.0797	4.34e-1	1.062
GAM	0.624±0.0815	0.219	0.629±0.0814	1.48e-5	0.595±0.0647	4.61e-2	1.056
Geometric	0.52±0.0624	8.69e-12	**0.519±0.062**	**6.54e-21**	0.52±0.0597	**9.98e-23**	0.9979
LDO	0.643±0.0812	0.847	0.641±0.0824	4.65e-1	0.62±0.0837	5.52e-1	1.035
MAGeCK	0.595±0.0481	6.85e-4	0.591±0.045	4.62e-1	0.591±0.0513	5.13e-1	**1.001**

Table S3 - Summary of proximity number bias (mean ± standard deviation) correction across methods in Project Score. Results are shown both for genome-wide (GW) and TP53-based proximity bias (i.e. wild-type (WT) and loss-of-function (LOF)). For genome-wide and TP53-based proximity bias, we also performed a *t*-test to assess whether each method's distribution is significantly different from the uncorrected one. For the TP53-based proximity bias, we computed the average ratio (AR) between the WT and LOF proximity bias scores for each method. Bold is the best outcome across methods.

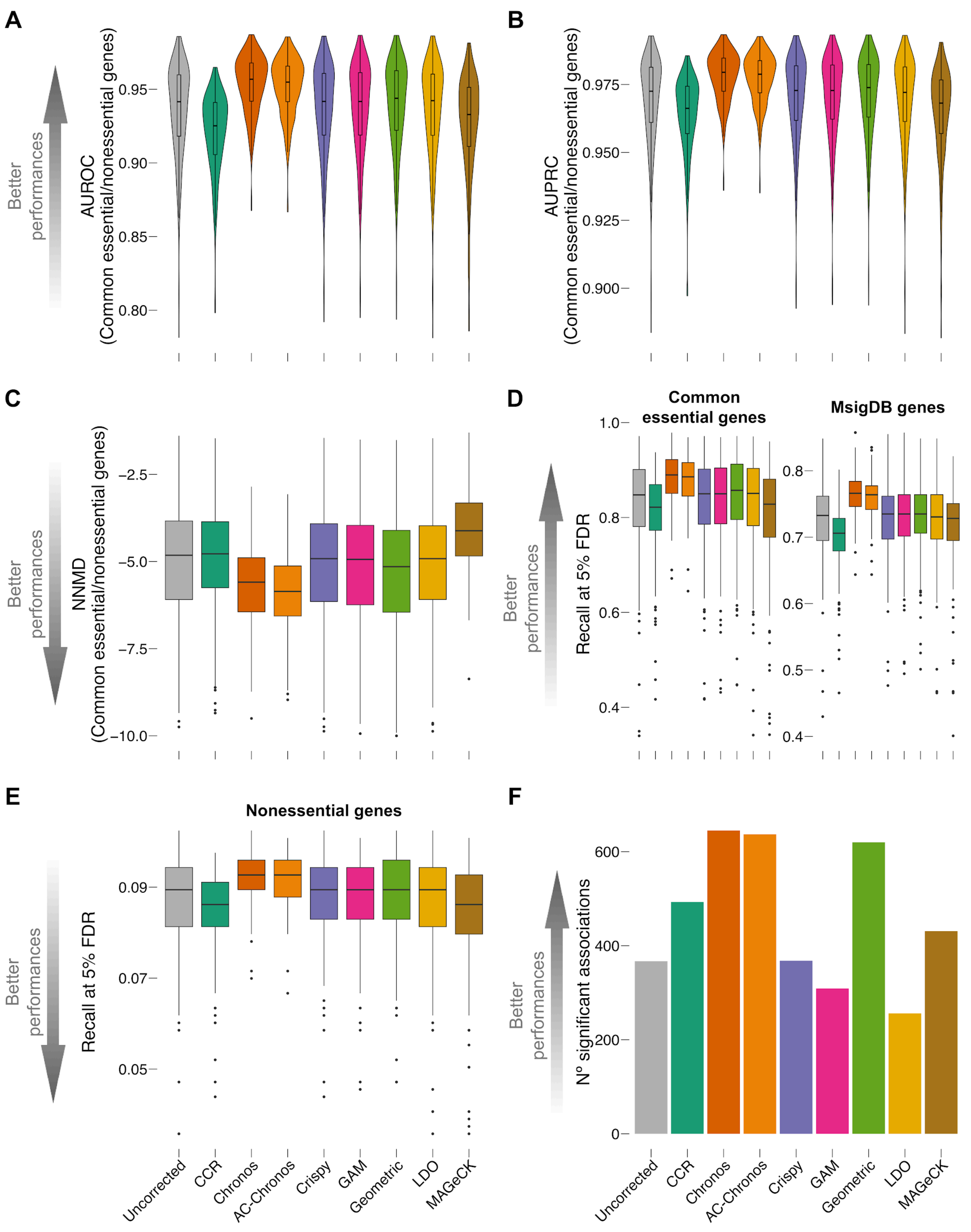

Fig. S4 - Assessment of data distortion and heterogeneity in Project Score. AB. Screen-wise area under the receiver operating characteristic (AUROC) curve (A) and area under the precision-recall curve (AUPRC) (B) were obtained when considering all the screens in Project Score (uncorrected version and processed with all tested methods) as rank-based classifiers of common-essential/non-essential genes, based on the depletion log fold change (LFC). C. Screen-wise null-normalised median difference (NNMD) between the distribution of LFCs of common essential and non-essential log fold change (LFC) distributions for the screens in Project Score (uncorrected version and processed with all tested methods). DE. Screen-wise recall at a 5% false discovery rate was obtained when considering each screen as in ABC but using different gene sets as positive controls (i.e. common essential genes, set of core-fitness genes from MsigDB (E) and non-essential genes (N)) for the Project Score dataset (uncorrected version and processed with all tested methods). F. Number of tissue-specific statistically significant (FDR < 5%) Cancer Functional Events (CFEs) / gene-essentiality associations identified in Project Score (unprocessed version and across correction

methods) via a systematic two-sided *t*-test. This test assesses differential gene essentiality (as measured by the gene depletion log fold-change (LFC)) across two sub-populations of cell lines stratified based on the presence/absence of the considered CFE.

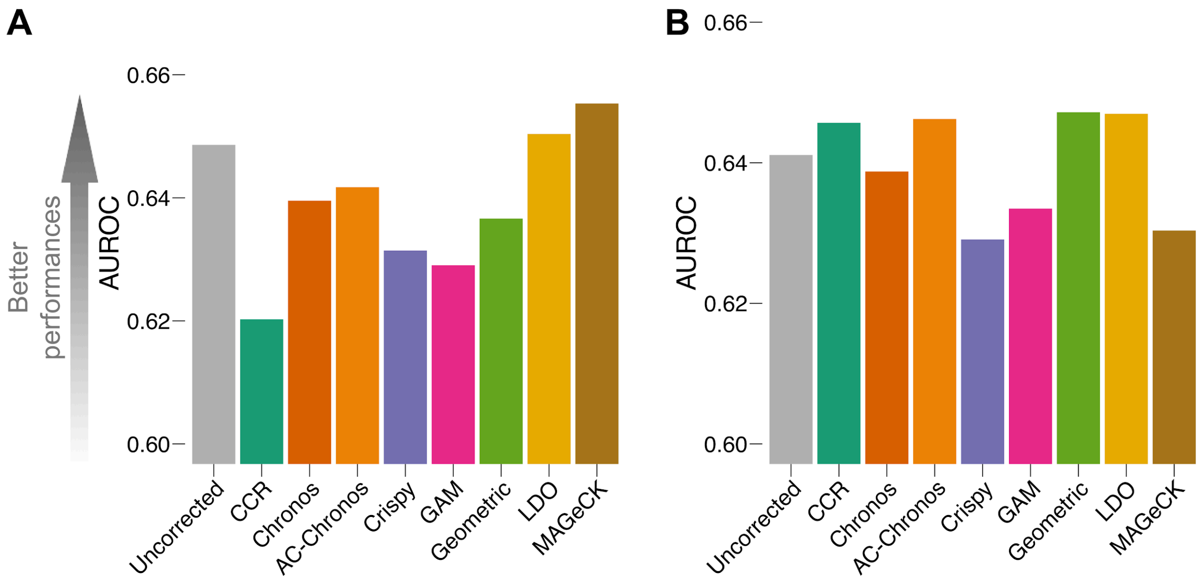

Fig. S5 - Identification of oncogenetic addictions. AB. AUROC obtained when considering all genes' LFCs scaled and pooled across screens in Project Achilles (A) and Project Score (B) (uncorrected version and processed with all tested methods) as a unique rank-based classifier of oncogenic addictions, and using cell line specific mutated oncogenes as positive controls and wild-type unexpressed (TPM < 1) oncogenes as negative controls.

Method	AUROC (common essential vs non-essential genes)	AUPRC (common essential vs non-essential genes)	NNMD (common essential vs non-essential genes)	AUROC (oncogenetic addictions)
Uncorrected	0.949±0.0399	0.972±0.0239	-5.19±2.02	0.649
CCR	0.93±0.0357	0.963±0.022	-4.7±1.67	0.62
Chronos	**0.961±0.0223**	**0.98±0.0116**	-5.93±1.53	0.64
AC-Chronos	0.96±0.0224	0.979±0.0117	**-6.09±1.59**	0.642
Crispy	0.949±0.0398	0.972±0.0237	-5.27±2.06	0.631
GAM	0.949±0.0397	0.972±0.0236	-5.3±2.07	0.629
Geometric	0.949±0.0406	0.972±0.0241	-5.41±2.15	0.637
LDO	0.949±0.0399	0.972±0.0239	-5.22±2.03	0.65
MAGeCK	0.953±0.0356	0.974±0.0211	-5.11±1.8	**0.655**

Table S4 - Summary of recall of common essential and non-essential genes and oncogenetic addictions (mean ± standard deviation) across methods in Project Achilles. Results are based on the following metrics: area under the receiver operating characteristic (AUROC), area under the precision-recall curve (AUPRC), null-normalised median difference (NNMD), and AUROC for oncogenetic addictions. Bold is the best outcome across methods.

Method	AUROC (common essential vs non-essential genes)	AUPRC (common essential vs non-essential genes)	NNMD (common essential vs non-essential genes)	AUROC (oncogenetic addictions)
Uncorrected	0.936±0.032	0.969±0.0164	-5.04±1.66	0.641
CCR	0.92±0.0273	0.964±0.0141	-4.91±1.55	0.646
Chronos	**0.954±0.0194**	**0.978±0.00902**	-5.74±1.08	0.639
AC-Chronos	0.952±0.0194	0.977±0.00904	**-5.91±1.05**	0.646
Crispy	0.937±0.0317	0.97±0.0159	-5.11±1.67	0.629
GAM	0.937±0.0315	0.97±0.0158	-5.15±1.68	0.633
Geometric	0.939±0.0312	0.971±0.0156	-5.32±1.77	**0.647**
LDO	0.936±0.032	0.969±0.0163	-5.1±1.67	**0.647**
MAGeCK	0.925±0.0352	0.964±0.0181	-4.12±1.14	0.63

Table S5 - Summary of recall of common essential and non-essential genes and oncogenetic addictions (mean ± standard deviation) across methods in Project Score. Results are based on the following metrics: area under the receiver operating characteristic (AUROC), area under the precision-recall curve (AUPRC), null-normalised median difference (NNMD), and AUROC for oncogenetic addictions. Bold is the best outcome across methods.

Method	Recall 5% FDR common essential genes	Recall 5% FDR MsigDB genes	Recall 5% FDR non-essential genes
Uncorrected	0.851±0.133	0.692±0.087	0.0756±0.0119
CCR	0.814±0.123	0.662±0.0802	**0.0723±0.011**
Chronos	**0.893±0.063**	**0.723±0.0402**	0.0793±0.00563
AC-Chronos	0.89±0.063	0.72±0.0395	0.079±0.00565
Crispy	0.853±0.131	0.695±0.0861	0.0758±0.0117
GAM	0.855±0.13	0.696±0.086	0.0759±0.0116
Geometric	0.856±0.132	0.694±0.0865	0.076±0.0118
LDO	0.852±0.133	0.693±0.0865	0.0757±0.0118
MAGeCK	0.866±0.118	0.702±0.0727	0.077±0.0105

Table S6 - Summary of recall at 5% false discovery rate of predefined gene sets (i.e. common essential, MsigDB and non-essential genes) and recall curves of amplified and amplified unexpressed genes. Results (mean ± standard

deviation) are computed across methods in Project Achilles. Bold is the best outcome across methods. Increased performances are defined as higher recall at 5% FDR for common essential and MsigDB genes, and lower recall at 5% for non-essential genes.

Method	Recall 5% FDR common essential genes	Recall 5% FDR MsigDB genes	Recall 5% FDR non-essential genes
Uncorrected	0.828±0.099	0.723±0.0556	0.0867±0.0104
CCR	0.807±0.084	0.698±0.0447	0.0844±0.00887
Chronos	**0.883±0.0518**	**0.766±0.0297**	0.0924±0.00549
AC-Chronos	0.878±0.0517	0.762±0.0288	0.0919±0.00546
Crispy	0.832±0.0952	0.727±0.0543	0.0871±0.01
GAM	0.833±0.0943	0.727±0.0542	0.0872±0.00991
Geometric	0.84±0.0909	0.728±0.0518	0.0879±0.00958
LDO	0.83±0.0993	0.724±0.0551	0.0869±0.0105
MAGeCK	0.805±0.105	0.717±0.0558	**0.0842±0.0111**

Table S7 - Summary of recall at 5% false discovery rate of predefined gene sets (i.e. common essential, MsigDB and non-essential genes) and recall curves of amplified and amplified unexpressed genes. Results (mean ± standard deviation) are computed across methods in Project Score. Bold is the best outcome across methods. Increased performances are defined as higher recall at 5% FDR for common essential and MsigDB genes, and lower recall at 5% for non-essential genes.

Method	Project Achilles	Project Score
Uncorrected	1740	367
CCR	**2240**	493
Chronos	1978	**645**
AC-Chronos	2135	637
Crispy	2116	368
GAM	2179	309
Geometric	1497	620
LDO	2009	256
MAGeCK	1805	431

Table S8 - Summary of the total number of significant associations between cancer functional events (CFEs) and strongly selective dependencies across methods in Project Achilles and Project Score. Bold is the best outcome across methods.
