# Supplementary material for: A benchmark of computational methods for correcting biases of established and unknown origin in CRISPR-Cas9 screening data
Source: Genome Biol. 2024 Jul 19;25:192. doi: 10.1186/s13059-024-03336-1 (PMC11264729; doi:10.1186/s13059-024-03336-1)
Supplement: Supplementary file 2 — Additional file 2. Review history. [file 13059_2024_3336_MOESM2_ESM.docx]

Review History

**First round of review**

**Reviewer 1**

**Were you able to assess all statistics in the manuscript, including the appropriateness of statistical tests used?**

**Yes:** All statistics in the manuscript could be assessed, including the appropriateness of statistical tests used.

**Comments to author:**

The study conducted by Vinceti and co-authors is a comprehensive comparison of methods for analyzing CRISPR-Cas9 screening data, that includes CRISPRcleanR, Chronos, Crispy, Local Drop Out (LDO), Generalised Additive Model (GAM), Model-based Analysis of Genome-wide CRISPR-Cas9 Knockout (MAGeCK), and the Geometric method. These methods were evaluated based on their ability to correct biases observed in large-scale CRISPR-Cas9 screening datasets. Two of these methods work unsupervised, CCR and LDO, while others, like Chronos, Crispy, GAM, MAGeCK, and Geometric, require additional data about the screened model. Each method implements different algorithms and modeling assumptions, building upon various hypotheses regarding the source of biases in CRISPR-Cas9 screening data. For bias correction, methods like MAGeCK and Chronos employ negative binomial distributions, while Crispy uses Gaussian process regression and GAM incorporates nonlinear dependencies. In terms of data input requirements, CCR and MAGeCK can accept input files in FASTQ or BAM format, while others generally require raw read count files. The Geometric method takes pre-processed sgRNA depletion LFCs as input. Notably, CCR and LDO work entirely unsupervised, processing CRISPR-Cas9 screening data without requiring additional information.
The benchmarking analysis utilized two large publicly available Cancer Dependency Map datasets to assess the performance of the methods in correcting biases and preserving true biological signals. Methods like GAM and CCR demonstrated better correction of copy number bias, aligning median gene LFCs towards zero across different copy number bins. Furthermore, the study evaluated proximity bias correction, where the Geometric method and CCR showed significant reductions, while Chronos exhibited increased proximity bias after processing. Finally, the assessment of data distortion revealed that most methods improved the detection of essential genes, oncogenic dependencies, and tissue-specific biomarkers associated with cancer dependencies compared to unprocessed data. In conclusion, the study highlights the importance of choosing appropriate techniques for analyzing CRISPR-Cas9 screening data to correct biases and preserve true biological signals.

Overall the study is timely, well executed, and to the extent that this reviewer can judge, comprehensive. However, given the fact that the group of the corresponding authors also authored one of the main algorithms used for benchmarking (CCR), it is hard to escape the slightly biased notion and argumentation for some of the conclusions about the tool's performance. For example, on page 11 (lines 54-55), it is stated that the difference in proximity between TP53 WT and LOF was reduced when using CCR or Geometric. This analysis however neglects that the total values are vastly smaller than compared to the other tested algorithms, and thus conclusions should be based on ratios rather than absolute differences. Despite this rather isolated but needed adjustment, it was a pleasure to review this manuscript.

**Reviewer 2**

**Were you able to assess all statistics in the manuscript, including the appropriateness of statistical tests used?**

**Yes:** no comments

**Comments to author:**

Vincenti et al describe a comparison of tools for analyzing data from genome-scale CRISPR knockout fitness screens. In these screens, gene-level fitness estimates are notoriously subject to bias due to copy number amplification, where a large number of CRISPR/Cas-induced double strand breaks can lead to locus-nonspecific cytotoxicity. The authors compare a variety of tools across a range of optimization metrics. The comparisons are reasonable and the conclusions are conservative.

The manuscript would benefit from a bit more focus in the conclusion on one key point. The methods include a range of supervised/unsupervised and single-/multi-screen analysis tools, with CCR and LDO being the only unsupervised, single-screen tools. It is expected that a supervised, multi-screen approach should outperform unsupervised, single-screen approaches and, if not, this should be cause for some discussion. Moreover, a more explicit comparison of CCR and LDO is justified, with CCR apparently trading sensitivity (e.g. Fig 3) for specificity (e.g. Fig 2). Figure 4 is a bit misleading, with there being almost no variation in the X-axes of Fig4B/C, making ranking on this axis of questionable relevance.

The analyses are robust and need no further work. The comparisons are very useful to the community, and with a bit more robust conclusions section, this paper should certainly be published.

**Authors Response**

**Point-by-point responses to the reviewers’ comments:**

Reviewer #1:
- The study conducted by Vinceti and co-authors is a comprehensive comparison of methods for analyzing CRISPR-Cas9 screening data, that includes CRISPRcleanR, Chronos, Crispy, Local Drop Out (LDO), Generalised Additive Model (GAM), Model-based Analysis of Genome-wide CRISPR-Cas9 Knockout (MAGeCK), and the Geometric method. These methods were evaluated based on their ability to correct biases observed in large-scale CRISPR-Cas9 screening datasets. Two of these methods work unsupervised, CCR and LDO, while others, like Chronos, Crispy, GAM, MAGeCK, and Geometric, require additional data about the screened model. Each method implements different algorithms and modeling assumptions, building upon various hypotheses regarding the source of biases in CRISPR-Cas9 screening data. For bias correction, methods like MAGeCK and Chronos employ negative binomial distributions, while Crispy uses Gaussian process regression and GAM incorporates nonlinear dependencies. In terms of data input requirements, CCR and MAGeCK can accept input files in FASTQ or BAM format, while others generally require raw read count files. The Geometric method takes pre-processed sgRNA depletion LFCs as input. Notably, CCR and LDO work entirely unsupervised, processing CRISPR-Cas9 screening data without requiring additional information.
The benchmarking analysis utilized two large publicly available Cancer Dependency Map datasets to assess the performance of the methods in correcting biases and preserving true biological signals. Methods like GAM and CCR demonstrated better correction of copy number bias, aligning median gene LFCs towards zero across different copy number bins. Furthermore, the study evaluated proximity bias correction, where the Geometric method and CCR showed significant reductions, while Chronos exhibited increased proximity bias after processing. Finally, the assessment of data distortion revealed that most methods improved the detection of essential genes, oncogenic dependencies, and tissue-specific biomarkers associated with cancer dependencies compared to unprocessed data. In conclusion, the study highlights the importance of choosing appropriate techniques for analyzing CRISPR-Cas9 screening data to correct biases and preserve true biological signals. Overall the study is timely, well executed, and to the extent that this reviewer can judge, comprehensive.

+ We thank the reviewer for their comprehensive summary of our work and for recognising its importance and relevance.


- However, given the fact that the group of the corresponding authors also authored one of the main algorithms used for benchmarking (CCR), it is hard to escape the slightly biased notion and argumentation for some of the conclusions about the tool's performance.

+ We understand the Reviewer's concerns about the possibility that some conclusions in our previous submission could have been slightly biased towards favouring our CCR method. However, we would like to respectfully highlight the solid, data-driven nature of those conclusions and the fair nature of our study, which we assure is free of any conflict of interest and solely aims at advancing the field in an open, transparent, fully reproducible, and collaborative manner.
As a matter of the fact, after reading the preprint of our study, our former collaborators at the Broad Institute informed us of a new version of their CRISPR data processing pipeline. Including this new pipeline in a revised version of our benchmark has led us to conclusions that differ significantly from those in the original version of our manuscript.

Specifically, we have now updated our analyses to include a new version of Chronos-corrected data, referred to as “arm-corrected (AC) Chronos” in the manuscript. Our collaborators implemented this to overcome the inability of the original Chronos to address the gene proximity bias. This updated analysis shows that AC-Chronos is the top-performing method for correcting both types of CRISPR-Cas9-associated biases, preserving data quality and heterogeneity. Consequently, AC-Chronos should be the method of choice when analysing multiple CRISPR screens jointly and when Copy-Number-Alteration (CNA) profiles are available for the screened models. Conversely, CCR, which is slightly outperformed by AC-Chronos, should be chosen when analysing individual or few screens and/or when CNA profiles of the screened models are not available.

To reflect these changes, we have updated all main and supplementary tables and figures (except Figure 1), edited our results, and included the following text in the conclusions of our manuscript:

Based on our results AC-Chronos should be the primary choice for the correction of CN and proximity biases as well as for its ability to minimise the impact on data quality, when processing multiple screens with available CN-information jointly. AC-Chronos also allows the identification of a larger number of total clinically relevant biomarker/dependency associations. Nonetheless, CCR offers very good performances for the correction of CN and proximity biases and its ability to work on a single-screen basis without any additional omic data makes it the method of choice in case of limited data availability.
On the other hand, the corrected datasets yielded by AC-Chronos and Chronos, which borrows information across screens, had a lower impact on the data quality and, thus, we were better able to recapitulate priori known common essential and non-essential genes.

- For example, on page 11 (lines 54-55), it is stated that the difference in proximity between TP53 WT and LOF was reduced when using CCR or Geometric. This analysis however neglects that the total values are vastly smaller than compared to the other tested algorithms, and thus conclusions should be based on ratios rather than absolute differences.

+ We agree with this observation and have now corrected the proximity bias comparison based on the TP53 mutational status by considering average ratios instead of the previous metric. Now, the two best-performing methods are AC-Chronos and Geometric and this is mentioned in the manuscript as it follows:

(...) AC-Chronos and Geometric not only yielded the largest reduction of average BMP across chromosome arms but were also able to minimise the difference of this value across TP53 wild-type and TP53 mutant cell lines (average ratio of 9.998 x 10-1 and 1.002 respectively, for Project Achilles and AR = 9.992 x 10-1 and 1.001 respectively, for Project Score).

- Despite this rather isolated but needed adjustment, it was a pleasure to review this manuscript.

+ We thank again the reviewer for their positive feedback.


Reviewer #2:
- Vincenti et al describe a comparison of tools for analyzing data from genome-scale CRISPR knockout fitness screens. In these screens, gene-level fitness estimates are notoriously subject to bias due to copy number amplification, where a large number of CRISPR/Cas-induced double-strand breaks can lead to locus-nonspecific cytotoxicity. The authors compare a variety of tools across a range of optimization metrics. The comparisons are reasonable and the conclusions are conservative.

+ We thank the reviewer for their positive comments.


- The manuscript would benefit from a bit more focus in the conclusion on one key point. The methods include a range of supervised/unsupervised and single-/multi-screen analysis tools, with CCR and LDO being the only unsupervised, single-screen tools. It is expected that a supervised, multi-screen approach should outperform unsupervised, single-screen approaches and, if not, this should be cause for some discussion.

+ As detailed in our response to Reviewer #1’s first comment, our revised manuscript now shows that the newly included and supervised arm-corrected (AC) version of Chronos is overall the best-performing in terms of both CNA- and proximity-bias correction and reduced impact on data quality (i.e. detection of core fitness genes or identification of biomarkers) when multiple screens are available.
However, the supervisedness nature of this tool relies on the strong assumption that CNAs are the only source of bias, thus causing potential limitations in its applicability to datasets where other sources of bias may be present (e.g. proximity bias or off-target effects). We now mention this aspect in the discussion:

On the other hand, we observed no clear distinction in performance between supervised (i.e. requiring CN or other kind of omics data) and unsupervised methods. A possible explanation is that CRISPR-Cas9 screening data are affected by multiple source of biases and supervised methods are designed to address only one (i.e. CN bias) or two (i.e. CN and proximity biases) of them, missing others such as off-target effects or differences in cellular responses.


- Moreover, a more explicit comparison of CCR and LDO is justified, with CCR apparently trading sensitivity (e.g. Fig 3) for specificity (e.g. Fig 2).

+ We now mention that although CCR works better than LDO in correcting CRISPR-Cas9-associated biases, it has a higher impact on data quality upon correction:

Of interest is the comparison between CCR and LDO as they stand as the only unsupervised single-screen methods. This becomes paramount when researchers are confronted with constraints such as a limited availability of CRISPR screens and an absence of additional omics data (e.g CN data). Notably, CCR demonstrates better ability over LDO in addressing CN and proximity biases associated with CRISPR-Cas9 screening data. Conversely, LDO exhibits a lower impact on data quality upon correction as elucidated by higher recall of common essential genes, while CCR shows a lower recall of nonessential genes, effectively trading sensitivity for specificity.


- Figure 4 is a bit misleading, with there being almost no variation in the X-axes of Fig4B/C, making ranking on this axis of questionable relevance.

+ We agree with the Reviewer and we have now removed the x-axes in Fig. 4B and C, showing only the data previously on the y-axes as bar plots.


- The analyses are robust and need no further work. The comparisons are very useful to the community, and with a bit more robust conclusions section, this paper should certainly be published.

+ We thank the Reviewer for these positive conclusive remarks. We have significantly extended our discussion and conclusion sections mentioning the updated analyses and the new results from AC-Chronos. We believe that, alongside the edits to the conclusions explicitly suggested by this Reviewer, these changes have increased the robustness of our study.

**Second round of review**

**Reviewer 1**

CRISPR-Cas9 dropout screens are powerful tools for studying biology by systematically knocking out genes in cancer cell lines to identify new cancer dependencies and therapeutic targets. However, data biases, including copy number amplifications (CN bias) and proximity bias (gene-independent responses from nearby loci), can confound results. Various computational methods aim to correct these biases. This study rigorously benchmarked seven methods on large datasets, evaluating their effectiveness in reducing CN and proximity biases and preserving data quality for detecting essential genes and cancer biomarkers. Results indicate AC-Chronos is superior for multi-screen analysis with CN data, while CRISPRcleanR excels in individual screens without CN data, with Chronos and AC-Chronos effectively identifying essential genes. The study offers guidance on selecting suitable bias-correction methods based on experimental conditions.

As to the originally raised concerns, the authors have cleared them all. Particularly the addition of the comparisons to the newly established AC-Chronos algorithm is positively noteworthy. Congratulations!

**Reviewer 2**

Authors have addressed my concerns with the initial version.
